# Assessment of health‐related behaviors among medical students: A cross‐sectional study

**DOI:** 10.1002/hsr2.1310

**Published:** 2023-06-06

**Authors:** Rabab G. A. El‐Kader, Rekha J. Ogale, Omar Omar Zidan, Omar Al Jadaan, Vijaya Kumardhas, Sirwan K. Ahmed, Kuldeep Dhama, Praveen SV, Mohammad Ebad Ur Rehman

**Affiliations:** ^1^ RAK College of Nursing RAK Medical and Health Sciences University Ras Al Khaimah United Arab Emirates; ^2^ Public Health & Community Medicine Department, Damietta Faculty of Medicine Al‐Azhar University Cairo Egypt; ^3^ Department of Pediatrics Rania Pediatric and Maternity Teaching Hospital Sulaymaniyah Iraq; ^4^ Department of Nursing University of Raparin Sulaymaniyah Iraq; ^5^ Division of Pathology ICAR‐Indian Veterinary Research Institute (IVRI) Bareilly Uttar Pradesh India; ^6^ Department of Management Studies National Institute of Technology Tiruchirappalli Tamil Nadu India; ^7^ Department of Medicine Rawalpindi Medical University Rawalpindi Pakistan

**Keywords:** health‐related behaviors, medical students, nutrition, physical activity, survey, tobacco, university

## Abstract

**Background and Aim:**

Students sometimes participate in harmful activities that adversely influence their behaviors and well‐being throughout college, which is one of the sensitive phases in an individual's life. *Aim*: To assess the health‐related behaviors of university students.

**Methods:**

A cross‐sectional study that includes systematic randomly selected 383 students from various colleges of Ras Al Khaimah Medical and Health Sciences University (RAKMHSU), Ras Al Khaimah Emirate, United Arab Emirates. A self‐reported questionnaire included students' demographic traits and behaviors, including safety, medication intake, cigarette smoking, nutrition, physical activity, and health‐related topics.

**Results:**

Most participants were females (69.7%), 13.3% were obese while 28.2% were overweight. The data revealed a significant difference between male and female students regarding medication intake without prescription, nutrition, physical activity, and health‐related topics. The data also revealed that the majority of the students were attempting to lose weight, and the former male smokers had fewer trials to quit the use of all tobacco products than females.

**Conclusion:**

More than a quarter of participants were overweight, and the majority of students did not adhere to the guidelines for safety and nutritious eating. This study recognized significant health promotion opportunities for university students that can be carried out to establish a healthier youth for society.

## 1 INTRODUCTION

A changeover from secondary school to university education is a critical period in student's life. The students are more likely to participate in unhealthy practices that could have a long‐term negative impact on their health, such as lack of exercise, and poor eating habits. One of the most crucial determinants influencing the maintenance and improvement of health is health‐related behaviors and lifestyle.^1^, ^2^

University life is full of challenges for young adults. It becomes more demanding as they face various cognitive and emotional changes grounded in biological, psychological, social, and economic forms. Young people confront several demands when they enroll in college that include staying away from home, adjustment to a new multiethnic and multicultural environment, new food types and patterns, independent living, finding new friends, and still trying to be self‐reliant, coping with higher‐level studies, and academic stresses.^3^ Additionally, young people frequently exhibit curiosity and exploration, exposing them to various health risks.^4^

On the other hand, colleges and universities may play a significant role in lowering the prevalence of overweight in the adult population through the promotion of good nutrition and exercise guidelines to apply for the healthy weight management program. So, many college students may experience changes in the pattern of their lifestyle during their college years.^5^ The healthy lifestyle of young adults, which may establish during college, significantly influences their health and, also, their prospective families. Additionally, an unhealthy lifestyle of college students may lead to unfavorable physiological consequences in their future. So, college students are in a life stage and situation where it is easier for them to change their behavior.^6^ Few large‐scale studies conducted at the university levels in numerous low‐, middle‐, and high‐income nations have discovered that there were relatively few people practicing healthy eating habits,^7^ presence of depressive symptoms,^8^ physical inactivity,^9^ involvement in gambling and high rate of overweight or obesity among students at the college.^10^

In the United Arab Emirates (UAE), an Arab nation with a high standard of living, noncommunicable diseases (NCDs) are to blame for 77% of fatalities.^11^ Rahim et al.^12^ reported that in the Arab world, including the UAE, there is an increase in the prevalence of NCDs, diabetes, cancer, chronic lung illnesses, and cardiovascular disease. Indicators of behavioral NCD health risk are particularly prevalent among Arab children and adults. Hence, this study aimed to assess medical university students' health behavior in relation to safety, smoking, nutritional habits, and physical activities to know what may affect their health. Research results may be beneficial for planning and organizing university health promotion activities to improve the health status of young adults who represent a significant community investment.

## 2 METHODS

### 2.1 Research design and study population

This research is a descriptive survey study based on a cross‐sectional approach. The participants were students from Ras Al Khaimah Medical and Health Sciences University (RAKMHSU) during the academic year 2019–2020. The population under study was undergraduate students studying at four colleges of RAKMHSU viz., Medical, Dental, Pharmacy, and Nursing. The total population size was 1085, and the distribution in four colleges was as follows: Doctor in Medicine (MD) (449), Bachelor of Dental Surgery (BDS) (343), Bachelor of Pharmacy Program (BPharm) (171), and Bachelor of Science in Nursing Program (BSN) (122). Students who reported the presence of a physical illness (e.g., heart disease or bronchial asthma) at the time and those who declined to take part in the study were not included.

### 2.2 Sampling technique and sample size calculation

The students were chosen using a systematic random sampling approach within each different academic year in all the four colleges. The sample size was determined by taking the frequency of unhealthy behaviors among the students as 47% to the estimated proportion of students.^13^ The significance level (*p* = 0.05) and intent of error was 5% (deviation from the actual value).

With a 47% proportion at a 95% confidence interval, the estimated sample size calculated was 383. A total of 383 students were finally surveyed.

### 2.3 Data collections and tools

A lifestyle questionnaire was adapted from National Youth Risk Behavior Survey, 2017.^14^ It included two parts, Part 1: questions about students' demographic characteristics which included age, gender, nationality, college, academic year, previous year's academic performance, family history of lifestyle diseases, weight, and height. Part 2: assessment of student health‐related behavior; safety, medication intake, cigarette smoking, food consumption, physical activity, body weight perception, and health‐related topics. Necessary modifications were made to make it suitable for the situation of lifestyle in UAE. Modifications were made based on the experience of the researcher and the consultation of experts working in this field. The instrument's content validity was entrenched by an extensive literature review on the topic, along with the endorsement given by experts from the nursing and medical fields.

A self‐administered questionnaire was distributed to the students in the presence of the faculty. The researchers introduced themselves, explained the aim of the study and the researchers obtained the anthropometric measurements. The survey took approximately 15 min per student to be completed.

### 2.4 Pilot study

Thirty students participated in the pilot study (not included in the study) to test the questionnaire to ensure its suitability for data collection. Minor adjustments to the questionnaire were modified.

### 2.5 Measurements

The digital electronic Seca 284 were used to measure the body's weight and height, respectively. Body mass index (BMI), which measures a person's weight in kilograms in relation to their height in square meters, was used to determine their current weight status. The person was weighed to the nearest 0.1 kg while wearing light clothing, having all of his or her pockets empty, and going barefoot. To the nearest 0.1 cm, the subject's height was measured while standing barefoot. Categories of BMI were utilized to determine weight status. On the basis of Center for Disease Control and Prevention^15^; students were categorized into four groups, according to their BMI: underweight (BMI = 18.5), normal weight (BMI = 18.5–24.9), overweight (BMI = 25–29.9), and obese (BMI = 30).

### 2.6 Ethical considerations

Formal approval was obtained for this study from Ras Al Khaimah Medical and Health Sciences University, Research and Ethics Committee (RAKMHSU‐REC) with reference number: 150‐2019‐F‐N. The participants were explained about the process of research and data collection. They were told that there was no requirement for names or IDs and the participation was entirely voluntary. On their acceptance, they were asked to sign the consent form. They were assured of the anonymity and confidentiality of the collected information. The participants had the right to discontinue their participation at any moment and without providing a reason. The data was only collected, entered, and analyzed under strict confidentiality, and it was solely intended for scientific purposes.

### 2.7 Data analysis

The collected questionnaires were examined for accuracy and logical coherence. After data were coded and entered on an Excel‐based data sheet (Microsoft Corporation 2018). The statistical analysis was performed using SPSS (IBM Corp. Released 2012. IBM SPSS Statistics for Windows, Version 21.0; IBM Corp). Descriptive and inferential statistics were generated; for quantitative variables, mean and standard deviation were determined; for categorical variables, number and percentage were computed. Analytical statistics were performed using *χ* ^2^. At a *p* value of 0.05 or less, differences were deemed statistically significant.

## 3 RESULTS

**Figure 1:**
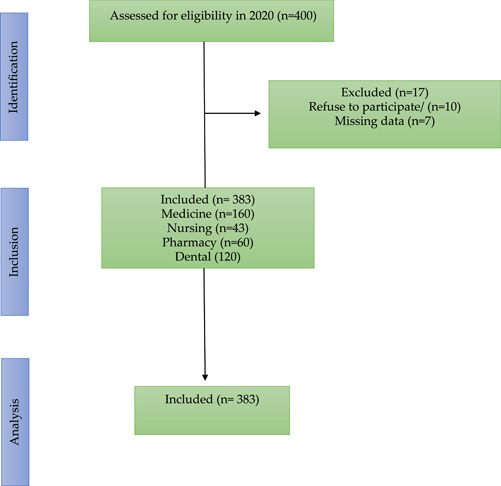
STROBE flowchart.

Out of the 400 students who were invited to participate in the study, 7 out of 400 were excluded due to missing data, 10 out of 400 refused to participate. Finally, 383 students successfully completed the questionnaire, resulting in an impressive response rate of 95.7% (Figure 1). Their ages ranged from 17 to 36 years, with a mean and standard deviation of 22.7 (6.4). The majority of the students were females (69.7%). According to their BMI interpretation, 55.1% of the students had normal weight, 28.2% were overweight, and 13.3% were obese. Less than half (41.8%) of the students were enrolled in medical college, and most of the students were either in their first or second‐year class (59%). Around half (47.5%) of the students were Arab. Almost all of the participants (98%) had a family history of chronic disease as diabetes (50.9%), hypertension (40.7%), cardiac diseases (19.3%), cancer (17%), and COPD (4.2%). More than one‐fifth (27.2%) of the students reported having families with smoking status, and 40.7% of the students stated their academic grades as mostly B (Table 1).

**Table 1:** Demographic data of the students (*n* = 383).

Demographics	Frequency	%
*Age (years)*		
<20	122	31.8
20–<30	212	55.4
≥30	49	12.8
Mean (SD)	22.75 (6.46)	
*Sex*		
Male	116	30.3
Female	267	69.7
*College*		
Medicine	160	41.8
Nursing	43	11.2
Pharmacy	60	15.7
Dental	120	31.3
*Class*		
First	122	31.8
Second	104	27.2
Third	49	12.8
Fourth	77	20.1
Fifth	31	8.1
*Nationality*		
Local	72	18.8
Non‐Arab	129	33.7
Arab	182	47.5
*Family history of chronic diseases*		
Yes	383	100
*Body mass index*		
Underweight	13	3.4
Normal weight	211	55.1
Overweight	108	28.2
Obesity	51	13.3
*Smoking status of family*		
No	279	72.8
Yes	104	27.2
*Academic standing*		
A majority of A's	106	27.7
A majority of B's	156	40.7
A majority of C's	92	24.0
A majority of D's	22	5.8
A majority of F's	7	1.8

The differences in health practice among male and female students are displayed in Table 2. The study findings highlight that very few students, both females (15%) and males (12.1%), sometimes wore seat belts when riding in a car driven by someone else with no significant difference among males and females (*p* = 0.30). Concerning medication intake, 48.1% of females compared with 65.5% of male students use of prescription painkillers without a doctor's prescription or otherwise outside of what a doctor advised differed significantly between male and female students (*p* = 0.000).

**Table 2:** Students health practice in relation to their gender.

Variables	Female (267)					Male (116)					*p* value
	Never	Rarely	Sometimes	Often	Always	Never	Rarely	Sometimes	Often	Always	
*Safety*											
‐When riding in a vehicle that is being operated by someone else, buckle up.	147 (55.1)	29 (10.9)	40 (15)	46 (17.2)	5 (1.9)	72 (62.1)	8 (6.9)	14 (12.1)	22 (19)	0	0.308
‐Text or e‐mail while operating a car or other vehicle	205 (76.8)	33 (12.4)	13 (4.9)	10 (3.7)	6 (2.2)	86 (74.1)	12 (10.3)	2 (1.7)	13 (11.2)	3 (2.6)	0.076
Medication intake											
Take prescription pain medicine^a^	11 (4.1)	96 (36)	18 (6.7)	13 (4.8)	128 (48.1)	3 (2.6)	16 (13.8)	10 (8.6)	11 (9.5)	76 (65.5)	0.000
Anyone offered, sold, or provided you an illicit drugs	259 (97)	8 (3)	0	0	0	109 (94)	7 (6)	0	0	0	0.132FET
Nutrition											
Drink 100% fruit juices	70 (26.3)	45 (16.9)	81 (30.3)	47 (17.6)	23 (8.9)	38 (32.7)	12 (10.3)	40 (34.5)	23 (19.8)	3 (2.6)	0.309
Eat fruits	31 (11.6)	23 (8.6)	81 (30.3)	70 (26.3)	62 (23.2)	15 (12.9)	4 (3.4)	57 (49.1)	10 (8.6)	30 (25.8)	0.000
Eat green salad	31 (11.6)	23 (8.6)	81 (30.3)	70 (26.3)	62 (23.2)	15 (12.9)	4 (3.4)	57 (49.1)	10 (8.6)	30 (25.8)	0.000
Take a can, bottle, or glass of soda and sports drink	9 (3.4	63 (23.6)	50 (18.7)	24 (9.0)	121 (45.3)	7 (6.0)	23 (19.8)	29 (25)	7 (6.0)	50 (43.1)	0.194
Eat food high in fat, saturate fat and cholesterol	3 (1.1)	7 (2.6)	14 (5.2)	9 (3.4)	234 (87.6)	3 (2.6)	6 (5.2)	7 (6.0)	100 (86.2)	0	0.289
Sip on some simple water	21 (7.9)	10 (3.7)	14 (5.2)	13 (4.8)	209 (78.2)	3 (2.6)	0	8 (6.9)	6 (5.2)	99 (85.3)	0.000
Drink milk	85 (31.8)	19 (7.1)	70 (26.3)	35 (13.1)	58 (21.7)	27 (23.3)	3 (2.6)	38 (32.7)	17 (14.6)	31 (26.7)	0.042
Eat breakfast	39 (14.6)	22 (8.2)	50 (18.7)	39 (14.6)	117 (43.8)	19 (16.4)	1 (0.9)	22 (8.2)	39 (14.6)	45 (38.7)	0.032
Physical activity											
Physically active^b^	72 (27)	17 (6.4)	105 (39.3)	49 (18.3)	24 (8.9)	32 (27.6)	6 (5.2)	42 (36.2)	17 (14.6)	19 (16.4)	0.240
Doing exercises^c^	10 (3.7)	24 (9)	79 (29.5)	31 (11.6)	123 (46.1)	57 (49.1)	7 (6)	15 (5.6)	12 (10.3)	25 (21.5)	0.005
Play in sports teams	199 (74.5)	30 (11.2)	26 (9.7)	6 (2.2)	6 (2.2)	80 (69)	9 (7.8)	19 (16.4)	5 (4.3)	3 (2.6)	0.133
Watch TV during the university day	105 (39.3)	57 (21.3)	87 (32.6)	6 (2.2)	12 (4.5)	33 (28.4)	34 (29.3)	37 (31.8)	9 (7.7)	3 (2.6)	0.005
Health‐related topics											
Use a sunburn applications	192 (71.9)	20 (7.5)	41 (15.3)	6 (2.2)	8 (3.0)	88 (75.9)	16 (13.8)	8 (6.9)	1 (0.86)	2 (1.7)	0.204
Visit a dentist for a check‐up	21 (7.8)	16 (6.0)	45 (16.8)	50 (18.7)	135 (50.6)	18 (15.5)	6 (5.2)	14 (12.1)	30 (25.8)	48 (41.4)	0.000
Take adequate sleep per day	0	16 (6.0)	25 (9.4)	145 (54.3)	81 (30.3)	0	2 (1.7)	13 (11.2)	47 (40.5)	54 (46.5)	0.010

^a^ Use prescription painkillers differently than prescribed by a doctor or without a prescription.

^b^ Be physically active for at least 60 min each day.

^c^ Performing muscle‐strengthening or muscle‐toning exercises, such as pushups, sit‐ups, or weightlifting.

There were significant disparities between males and females in their daily consumption of fruits, green salad, breakfast, milk, and an adequate amount of water per day (*p* = 0.000, 0.032, 0.042, and 0.000, respectively). Less than half (45.3%) of female students always drank a can, bottle, or glass of soda and sports drink per day opposed to 43.1% of male students, with no significant difference (*p* = 0.194) among them. The majority of male and female students (86.2%) (87.6%) ate food high in fat and cholesterol, with no significant difference (*p* = 0.289) among both genders.

In terms of physical activity, 46.1% of female compared with 21.5% of male students always perform pushups, sit‐ups, or weightlifting activities with a statistically significant difference (*p* = 0.005). But when it came to participating in sports teams and being physically active for at least 60 min/day, 39.3% of females compared with 36.2% of male students sometimes do it with no discernible difference between male and female students (*p* = 0.240, 0.133, respectively). Less than one‐third (32.6%) of female students and 31.8% of male students sometimes watched TV during their university days, with significant differences (*p* = 0.005).

Analyzing the health‐related topics, 50.6% of females compared with 41.4% male students visiting a dentist for a check‐up with a statistically significant differences were identified between males and females (*p* = 0.000). In relation to sleeping adequate time per day, 30.3% of female and 46.5% of male students always have adequate sleep with a statistically significant difference (*p* = 0.010). However, 71.9% of female and 75.9% of male students never use of an anti‐sunburn product with no discernible difference between male and female students (*p* = 0.204) (Table 2).

Table 3 illustrates the experimentation of the students with tobacco products, 16.5% of females compared with 44.0% of male students had undergone a trial of cigarette smoking with a statistically significant difference (*p* = 0.000). Notably, the average age of first‐time testing for all forms of tobacco was 14–15 years among females compared with 11–12 years of age among males with a statistically significant difference (*p* = 0.008). On average, in the last 30 days, the female students smoked cigarettes for 10 days, whereas the males smoked for all 30 days with a statistically significant difference (*p* = 0.001). Furthermore, the average number of days students used chewing tobacco, snuff, dip, or dissolvable tobacco products and smoked cigars, cigarillos, or small cigars was 1 day among female students during the course of the last 30 days, whereas it was 2 days among males with a statistically significant difference (*p* = 0.000). Male students smoke more cigarettes than females per day with no statistically significant difference (*p* = 0.082). Half (50%) of female and 33.3% of male students had undertaken a trial to quit the consumption of tobacco products with no statistically significant difference (*p* = 0.052).

**Table 3:** Experimentation with tobacco products and tobacco use patterns of the smoking students.

Variables	Female (267)		Male (116)		*p* value
	Frequency	%	Frequency	%	
Experimentation of cigarette smoking, even one or two puffs	44	16.5	51	44.0	0.000
Average age of first trial (years)	14–15		11–12		0.008
Average daily cigarette consumption over the last 30 days	10 days		30 days		0.000
Average number of cigarettes smoked per day	2 cigarettes		9 cigarettes		0.001
Average daily usage of tobacco products during the last 30 days, including chewing, snuff, dip, snus, and dissolvable	1 day		2 days		0.000
The average number of days in the past 30 days that you have smoked a cigar, cigarillos, or small cigars	1 day		1 day		0.082
Trial to stop using all tobacco products	22	50%	17	33.3%	0.052

Table 4 describes the students' perception of their weight. The majority of the students (76.2%) expressed that they had the right weight or were slightly overweight. In spite of this, 58.7% of the students wanted to lose weight, whereas only 12.8% stated that they were very overweight, while another 11.7% they did not want to make any efforts to lose weight.

**Table 4:** Body weight perception of the students (*N* = 383).

	Frequency	%
*Description of body weight*		
Right weight	157	41.0
Slightly overweight	135	35.2
Slightly underweight	37	9.7
Very overweight	49	12.8
Very underweight	5	1.3
*Attempts for body weight*		
Gain weight	28	7.3
Not trying to do anything	45	11.7
Lose weight	225	58.7
Stay the same weight	85	22.2

## 4 DISCUSSION

A healthy lifestyle is the cornerstone of the overall health of the people, and healthy people determine the health of the nation. Today's youth are the greatest strength of the nation. Future health of university students might be affected by their current dietary habits, exercise habits, and lifestyle choices. The current study highlights the health‐related behaviors among medical university students in RAK Emirate, UAE. The findings showed that male and female students' medicine intake varied significantly. It was identified that about half of the students were using prescribed painkillers either differently than a doctor had advised or without a doctor's prescription. This is consistent with research by Alduraibi and Altowayan^16^ in Saudi Arabia, who reported that the majority of students (88%) chose painkillers as their primary form of self‐medication, accounting for more than half (64%) of students who engaged in this activity in the past 6 months. Moreover, Alshahrani et al.^17^ in Saudi Arabia, and Alshogran et al.^18^ in Jordan, reported that for a variety of symptoms, the majority of the student's used self‐medication.

It is a well‐known fact that adventures and risk‐taking are the features of youngsters which may sometimes be damaging to their health. This study reported that most of the students never used a seatbelt while riding in a vehicle that was being driven by someone else. The university students are relatively young and do not consider the importance of fastening the seat belts while riding in a car for their safety.^19^ Similar findings were reported by Fildes et al.^20^ that the likelihood of seatbelt use among teenagers and young adults is lower.

Physical activity is one of the most crucial factors affecting health. The results showed a substantial difference between males and females in terms of the degree of physical activity among students; more female students engaging in regular muscle‐building exercises than male students. This finding may be attributable to the students’ improved health awareness, which is seen in a positive relationship between their exercise activity and it. More so, constant motivation is required to perform regular physical activities, especially to maintain body weight and health. Similar findings were reported by various studies carried out among college students. Almutairi et al.^2^ stated that there were significant differences across response waves of doing stretching exercises at least three times per week among the students in nonhealth and health‐specific colleges. However, male and female students responded to being physically active for at least 60 min each day in the same way, with no discernible differences. Klainin‐Yobas et al.^21^ reported that nursing students' overall physical fitness levels ranged from poor to moderate in Thailand, with the lowest scores for the components of body flexibility and cardiovascular fitness. Almutairi et al.^2^ and Park et al.^22^ in Korea, stated that the students deny and do not engage in light to moderate physical activity.

With regard to nutrition and food habits, there was a significant difference in daily intake of nutritious food between male and female students. Consumption of fruits and green salad on a regular basis was reported by only one‐fourth and less than one‐fourth of the male and female students, respectively, whereas a substantial number of male and female students said they eat fruits and green salad only sometimes. This is in agreement with the results of previous studies, who reported that most students have a low intake of fruits and vegetables.^2^, ^23^, ^24^, ^25^ In contrast to these findings, Sakamaki et al. (2005)^26^ reported higher consumption of fruits and vegetables among university students in China. The present study has shown that much less than 50% (44% females and 39% males) of the students were eating breakfast, which is in contrast with the result of a study by Al‐Rethaiaa et al.^26^ and Almutairi et al.^2^, ^27^ who reported that the immense majority of students eat breakfast.^2^, ^19^ The results of this study exhibited a significant difference between male and female students in relation to drinking milk and adequate amounts of water. This is in line with Almutairi et al.^2^ reported that more than half of the students had two to three servings of milk, yogurt, or cheese each day.

In the current study, most of the students ate foods that were rich in fat and cholesterol. This result is in agreement with other studies concluded that majority of the students did not select diets reduced in fat, saturated fat, and cholesterol.^2^, ^27^, ^28^ This may be a result of external influencing factors such as classmates, easy availability of junk food, the taste of the food, and the campus setting where students spend a lot of time together. However, despite being aware of good eating habits, students continue to favor high‐sugar and fat items.

Investigating the health‐related practices of the students, significant differences were identified between males and females in relation to visiting a dentist for a check‐up and sleeping adequate time per day (*p* = 0.000, 0.010, respectively). However, there was no discernible difference in the use of sunburn remedies between male and female students (*p* = 0.204). This is in line with Tsai and Li,^29^ who reported the correlation between sleep qualities and rise time, time in bed, and sleep efficiency to be stronger in male college students than in female.^30^ This is in contrast to a study's findings by Gallego‐Gómez et al.^30^ who stated that the nursing students (30.4%) had bad sleep habits.

The results of the existing study have exposed that substantially a smaller number of female (16.5%) compared with male (44.0%) students had experimented with tobacco products. The average number of cigarette smoking days during the past 30 days was 10 among females and 30 among males. Male students smoke more cigarettes than females per day. Half of the females, compared with 33.3% of male students, had a trial to quit the used tobacco products. This is in consensus with a study by Chinwong et al.,^31^ in Thailand, who concluded that male and female university students differed in smoking behaviors and intention to quit.^31^ Male students had higher smoking behavior, and they were more likely to smoke every day with a high average number of cigarettes daily, and the females were more likely than males to express intention to quit in the next 30 days. Hafez et al. in Egypt also reported that the smoking prevalence is significantly higher among male university students.^32^

Maintaining body weight is an important aspect of healthy living. The present study has highlighted that about half of the students were attempting to lose weight, compared with one‐third of students who identified as overweight or obese. This is in accordance with a study by Asfour et al.^33^ in UAE, more persons than those who reported being overweight attempt to lose weight. People are becoming increasingly aware of their body weight as overweight and obesity are the leading causes of NCDs in today's world and can be linked to behavioral practices including poor eating habits, inactivity, stress, and unhealthy habits like smoking. This risky behavior must be addressed by individuals, health educators, and healthcare authorities on a priority basis.

Health science students are the future healthcare professionals who will impart health teaching to individuals and communities on healthful living. Therefore, it becomes most important for them to learn and practice healthy behavior and be an exemplar modeling healthy lifestyles to the other people.

Universities are perfect locations/platforms for applying for goal‐oriented health promotion programs, planning and conducting those programs to motivate students to be more responsible for their own health as well as practice healthy behaviors with the aim of preventing diseases and promoting health are of paramount significance. Additionally, universities must develop curricula and counseling programs with the goal of providing students the information, encouragement, and empowerment required to make wise decisions about their health.

### 4.1 Limitation

The university students who provided the data may have provided responses that they believed the researchers wanted to hear because the data gathering method relied on self‐reporting. Furthermore, because only one university and only health science students was included in the survey, the findings cannot be applied to university students in the entire UAE.

## 5 CONCLUSION

In conclusion, more than one‐quarter of the participants in the present study were overweight, and most of the students were less adherent to recommendations regarding to the use of safety belt and text or e‐mail while operating a car. There was a significant difference between male and female students regarding medication intake without prescription. The analysis showed a significant difference between male and female students regarding the elements of nutrition, physical activity, and health‐related topics. The majority of the students, according to the data, were trying to slim down, and former smokers of tobacco products who were males had less success quitting than those who were females.

## AUTHOR CONTRIBUTIONS

**Rabab G. A. El‐Kader**: Conceptualization; data curation; formal analysis; funding acquisition; investigation; methodology; project administration; resources; validation; visualization; writing—original draft; writing—review and editing. **Rekha J. Ogale**: Conceptualization; data curation; formal analysis; funding acquisition; investigation; methodology; project administration; software; validation; visualization; writing—original draft; writing—review and editing. **Omar Omar Zidan**: Conceptualization; data curation; formal analysis; investigation; methodology; project administration; resources; software; validation; visualization; writing—original draft; writing—review and editing. **Omar Al Jadaan**: Conceptualization; data curation; formal analysis; investigation; methodology; project administration; resources; software; validation; visualization; writing—original draft; writing—review and editing. **Vijaya Kumardhas**: Conceptualization; data curation; formal analysis; investigation; methodology; project administration; resources; software; validation; visualization; writing—original draft; writing—review and editing. **Sirwan K. Ahmed**: Data curation; formal analysis; funding acquisition; investigation; methodology; project administration; resources; software; validation; visualization; writing—original draft; writing—review and editing. **Kuldeep Dhama**: Data curation; formal analysis; funding acquisition; investigation; methodology; project administration; resources; software; supervision; validation; visualization; writing—review and editing. **Praveen SV**: Methodology; resources; software; validation; visualization; writing—review and editing. **Mohammad Ebad Ur Rehman**: Resources; visualization; writing—review and editing.

## CONFLICT OF INTEREST STATEMENT

The authors declare no conflict of interest.

## ETHICS STATEMENT

All authors agree and provided their consent for submitting and publishing this work. This study was approved by RAKMHSU Research and Ethics Committee (RAKMHSU‐REC) with reference number: 150‐2019‐F‐N.

## TRANSPARENCY STATEMENT

The lead author Rabab G. A. El‐Kader affirms that this manuscript is an honest, accurate, and transparent account of the study being reported; that no important aspects of the study have been omitted; and that any discrepancies from the study as planned (and, if relevant, registered) have been explained.

## Data Availability

All data are available on request to the first author.
